# Pre-injury stimulant use in isolated severe traumatic brain injury: effect on outcomes

**DOI:** 10.1007/s00068-022-02095-7

**Published:** 2022-09-06

**Authors:** Dominik Andreas Jakob, Meghan Lewis, Elizabeth Robinson Benjamin, Tobias Haltmeier, Beat Schnüriger, Aristomenis Konstantinos Exadaktylos, Demetrios Demetriades

**Affiliations:** 1 Division of Trauma and Surgical Critical Care, Department of Surgery, Los Angeles County, University of Southern California Medical Center, University of Southern California, Los Angeles, CA 90033 USA; 2 Department of Emergency Medicine, Inselspital, University Hospital, University of Bern, Freiburgstrasse 16C, 3010 Bern, Switzerland; 3 Division of Trauma and Acute Care Surgery, Department of Surgery, Grady Health System, Emory University, Atlanta, GA 30327 USA; 4 Department of Visceral Surgery and Medicine, Inselspital, Bern University Hospital, University of Bern, Bern, Switzerland

**Keywords:** Amphetamine, Cocaine, Methamphetamine, Ecstasy, Trauma, Head

## Abstract

**Purpose:**

The aim of this study was to assess the impact of pre-injury stimulant use (amphetamine, cocaine, methamphetamine and/or ecstasy) on outcomes after isolated severe traumatic brain injury (TBI).

**Methods:**

Retrospective 2017 TQIP study, including adult trauma patients (≥16 years old) who underwent drug and alcohol screening on admission and sustained an isolated severe TBI (head AIS ≥3). Patients with significant extracranial trauma (AIS ≥3) were excluded. Epidemiological and clinical characteristics, procedures and outcome variables were collected. Patients with isolated stimulant use were matched 1:1 for age, gender, mechanism of injury, head AIS and overall comorbidities, with patients with negative toxicology and alcohol screen. Outcomes in the two groups were compared with univariable and multivariable regression analysis.

**Results:**

681 patients with isolated TBI and stimulant use were matched with 681 patients with negative toxicology and alcohol screen. The incidence of hypotension and CGS <9 was similar in the two groups.

In multivariable regression analysis, stimulant use was not independently associated with mortality (OR 0.95, 95% CI 0.61–1.49). However, stimulant use was associated with longer hospital length of stay (HLOS) (RC 1.13, 95%CI 1.03–1.24).

**Conclusion:**

Pre-injury stimulant use is common in patients admitted for severe TBI, but was not independently associated with mortality when compared to patients with negative toxicology. However, stimulant use was associated with a significant longer HLOS.

**Supplementary Information:**

The online version contains supplementary material available at 10.1007/s00068-022-02095-7.

## Introduction

The United States have some of the highest rates of drug use in the world [1]. According to the National Survey on Drug Use and Health (NSDUH) conducted in 2018 by the Substance Abuse and Mental Health Services Administration (SAMHSA), an estimated 53.2 million people aged 12 or older were illicit drug users, corresponding to 19.4% of the population. The use of illicit psychostimulants, such as cocaine and amphetamine-like agents (methamphetamine, ecstasy) in particular, have become an increasing problem. In North America, an estimated 2.1% of the adult population (6.9 million people) were estimated to have used cocaine in 2019. Similar numbers were reported for amphetamine, which was estimated at 2.3% (7.4 million people) and methamphetamine with about 1.8% of the population (nearly 5 million people). Ecstasy use has remained stable since 2015 at an estimated 2.5 million people (0.9%) [2]. There is evidence that the coronavirus disease (COVID-19) pandemic has aggravated the drug abuse problem in the United States. According to a web based survey of 5412 Americans in June 2020 one in 10 reported that they started or increased substance use because of the COVID-19 pandemic [3]. Another study published in June 2021 found increased amphetamine and 3,4-methylenedioxy methamphetamine (MDMA) positivity in trauma patients after stay-at-home order, but no difference in alcohol positivity [4].

Stimulant use is frequent in patients admitted for traumatic brain injury (TBI) because these drugs effect the central nervous system, and therefore contribute to a higher risk of accidents and violence [5–10]. In addition, stimulants activate sympathetic tone and affect the patient’s physiology. This can obscure the diagnosis in the emergency department, which may ultimately delay treatment and affect outcomes.

In response to the growing prevalence, several studies have examined the relationship between stimulant use [11–16], injury patterns, and patient outcomes. However, these studies have reported conflicting results regarding patient outcomes and stimulant use. It’s important to emphasize that most of these have studies included a general trauma population, which does not allow for a clean assessment, due to a large number of potential confounders.

In 2017, the American College of Surgeons (ACS) Trauma Quality Improvement program (TQIP) database provided, for the first time, detailed information concerning drug use among admitted trauma patients. This provides the basis for the following study, which evaluated simulant use among a large trauma population in the United States. To eliminate confounding factors, this study included only patients with isolated severe blunt head injury. We hypothesized that pre-injury stimulant use in isolated severe head injury is associated with worse outcomes compared to patients with no drug use.

## Methods

The study was approved by the Institutional Review Board of the University of Southern California.

### Patient selection and data collection

This retrospective study was performed using the ACS TQIP database from January 2017 to December 2017. The ACS TQIP database collects injury data from more than 750 trauma centers in the United States.

All adult trauma patients (≥16 years old) who underwent a drug and alcohol screening on admission were considered for this study. Patients with missing screening results for drug or alcohol, as well as patients with a drug screening only positive due to administered drugs for treatment were excluded from the study, to assess the significance of pre-injury substance use on outcomes. Finally, only patients with isolated severe head injuries (AIS ≥3) were extracted by excluding those with, face, neck, chest, abdomen, spine, extremity and external AIS ≥3.

In 2017, the TQIP database reported for the first time the presence of the following substances: amphetamine, barbiturate, benzodiazepines, cocaine, methamphetamine, ecstasy, methadone, opioid, oxycodone, phencyclidine, tricyclic antidepressant and cannabinoid without quantification. The urine toxicology reports only a binary result, positive or negative, depending on whether the threshold is met. Consequently, the present study analyzes the drug use as a binary variable. The blood alcohol level (BAL) is reported as a continuous variable with a value of 0.00 considered negative, and >0.00 as positive.

According to their physiological effects we categorized amphetamine, cocaine, methamphetamine and ecstasy as stimulants [17]. Patients with isolated stimulant use were subsequently defined as the detection of stimulants only in the toxicology report and a negative BAL. The control group included patients with no detection of illicit drugs in the toxicology report and a negative BAL.

Data collection included demographics (age, gender), comorbidities [current smoker, chronic renal failure, history of cerebrovascular accident (CVA), history of myocardial infarction (MI), hypertension, chronic obstructive pulmonary disease (COPD), diabetes mellitus, congestive heart failure, active cancer, mental disorder], mechanism and severity of injury, admission data [systolic blood pressure (SBP), heart rate (HR), GCS]. The alcohol screening variable and the BAL were reported, as well as the drug screening variable including the different drug information as mentioned above. Furthermore, diagnosis, procedure codes and time to procedures were collected. Hypotension was defined as a SBP <90 mmHg, and tachycardia was defined as a HR >120 beats per minute.

The primary outcome was in-hospital mortality. Secondary outcomes included craniectomy rate, time to craniectomy, mechanical ventilation requirements, overall complications, and hospital length of stay (HLOS). The following complications were recorded and summarized as overall complications: acute kidney injury, acute respiratory distress syndrome (ARDS), thromboembolic events including deep vein thrombosis (DVT) and/or pulmonary embolism (PE), severe sepsis, myocardial infarction, ventilator associated pneumonia (VAP), and stroke (CVA).

### Cohort matching

Due to the similar number of patients with stimulants use (*n* = 1055) and patients with no drug or alcohol use (*n* = 1323), a 1:1 cohort matching of patients under isolated pre-injury stimulant use and patients with no illicit drug or alcohol use was performed. For each patient with stimulant use, one control patient (no pre-injury drug or alcohol use) was matched on the basis of the following criteria: Age (>65, ≤65 years), gender, mechanism of injury (fall, motor vehicle collision (MVC), motor cycle collision (MCC), auto vs pedestrian (AVP), assault, others), head AIS and overall comorbidities. The matching tolerance was 0 for all matching criteria. Matching was performed without replacement.

### Statistical analysis

Normality of distribution was assessed using histograms, skewness, kurtosis, and the Shapiro–Wilk test. Univariate analysis was performed to identify differences in baseline and outcome variables between patients with pre-injury stimulant use and no drug or alcohol detection at all. Categorical variables were compared using Chi-square test or Fisher’s exact test. Fisher’s exact test was used when more than 20% of cells have expected frequencies <5 [18]. Mann–Whitney U test was used to compare medians for non-parametric continuous variables. Results were reported as numbers and percentages or medians and interquartile range (IQR).

In the matched cohort, a logistic regression analysis was used to examine independent risk factors associated with mortality, craniectomy, ventilation requirements, overall complications, and hospital LOS. Clinically important predictors (age, gender, mechanism of injury, overall comorbidities, admission HR, admission SBP, and head AIS) were correlated with the dependent variable using the Chi-square test or Fisher exact test, as appropriate, and entered in the regression models if the *p* value was less than 0.2. Not normally distributed dependent variables were log10 transformed for linear regression analysis. The regression coefficient (RC) and confidence interval (CI) were then back-transformed to the original scale for ease of interpretation.

Additional subgroup regression models were performed to account for any remaining differences in comorbidities despite extended matching. All comorbidities with a *p* < 0.2 between patients with pre-injury stimulant use and no drug or alcohol use (current smoking, chronic renal failure, history of CVA, hypertension and mental disorder) were included in the models. Furthermore, a regression analysis was performed in which patients under the influence of amphetamine/methamphetamine and cocaine were considered separately.

Results were reported as odds ratio (OR) and 95% CI or RC and 95% CI, as appropriate. The degree of multicollinearity between predictor variables was assessed using the variance inflation factor (VIF). A VIF <1.5 was assumed to exclude significant collinearity. Regression model performance was assessed using *χ*^2^ goodness of fit, Snell’s *R*-square, Nagelkerke *R*-square and area under the receiver operating characteristic (ROC) curve for logistic regression, *R*^2^ and adjusted *R*^2^ coefficients for linear regression.

Variables with *p* value <0.05 were considered significant. Statistical analysis was performed using SPSS for windows version 25.0 (SPSS Inc, Chicago, IL).

## Results

**Fig. 1:**
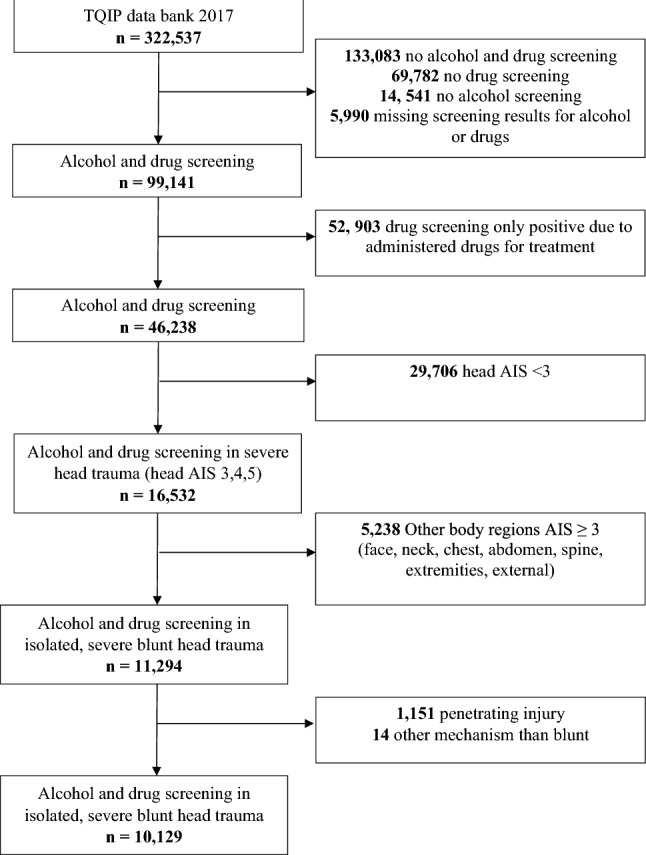
Patient Flowchart. *TQIP* Trauma Quality Improvement Program

Of 322,537 patients included in the 2017 TQIP database, 99,141 were screened for alcohol and drugs. Fifty-two thousand nine hundred and three patients were excluded because of a positive drug screening only due to administered drugs for treatment. Of the 46,340 remaining patients, 10,129 patients were identified with an isolated blunt severe head injury (head AIS ≥3) forming the basis of the present study (Fig. 1).

Of those, 8254 (81.5%) tested positive for at least one substance in urine toxicology. Overall, 2536 patients (25.0%) tested positive for polydrug use (≥2 substances). The most frequently detected substance was cannabinoid (4291 patients, 42.4%), followed by cocaine (1932 patients, 19.1%), amphetamine (1865 patients, 18.4%), benzodiazepine (1725 patients, 17.0%), opioids (1378 patients, 13.6%) and barbiturates (507 patients, 5.0%). Methamphetamine was detected in 471 patients (4.7%), oxycodone in 251 patients (2.5%), phencyclidine in 247 patients (2.4%), ecstasy in 177 patients (1.7%), and tricyclic antidepressant in 149 patients (1.5%).

Of all tested patients 3563 (35.2%) had a positive BAL. Overall, 2173 patients (61.0%) with a positive BAL also tested positive for at least another drug in the toxicology screening.

### Unmatched cohort characteristics

Finally, we identified 1055 patients under the exclusive influence of stimulants and 1323 patients with no drug or alcohol use at all.

Of the 1055 patients with pre-injury stimulant use, 439 (41.6%) tested positive exclusively for amphetamines, 413 (39.1%) for cocaine, 45 (4.3%) for methamphetamine and 5 (0.5%) for ecstasy. The remaining 153 (14.5%) patients tested positive for a combination of those drugs. The baseline characteristics, admission data, and injury characteristics as well as the outcomes of patients under the exclusive influence of stimulants compared to those with no drug or alcohol use is shown in Supplemental Tables 1–3.

### Cohort matching

A 1:1 cohort matching resulted in 681 matched cases in each group, which formed the basis of the present study. In the stimulant group 318 patients were positive only on amphetamine or methamphetamine, 263 patients only on cocaine, 5 patients only on ecstasy and 95 patients were positive for multiple stimulants.

All matching variables [age (>65, ≤65 years), gender, mechanism of injury (Fall, MVC, MCC AVP, assault, others), head AIS and overall comorbidities] were equally distributed between 681 patients under the exclusive influence of stimulants and 681 patients with no drug or alcohol use at all. As well, the ISS between the two groups was similar [16 (10–21) vs 16 (10–21), *p* = 0.547] (Table 1).

**Table 1:** Post matching baseline characteristics of patients with isolated use of stimulants compared to those negative for illicit drugs or alcohol

	All patients *N* = 1362 (%)	Stimulant only use *N* = 681 (%)	No drug/alc. use *N* = 681 (%)	*p-*value
Demographics				
Age*	47 (31–59)	47 (33–58)	48 (28–60)	0.475
≥ 65 years	188 (13.8)	94 (13.8)	94 (13.8)	1.000
Gender, male	1042 (76.5)	521 (76.5)	521 (76.5)	1.000
Mechanism of injury				
Fall	556 (40.8)	278 (40.8)	278 (40.8)	1.000
MVC	292 (21.4)	146 (21.4)	146 (21.4)	
MCC	60 (4.4)	30 (4.4)	30 (4.4)	
AVP	142 (10.4)	71 (10.4)	71 (10.4)	
Assault	184 (13.5)	92 (13.5)	92 (13.5)	
Other	128 (9.4)	64 (9.4)	64 (9.4)	
Comorbidities				
Overall	732 (53.7)	366 (53.7)	366 (53.7)	1.000
Current smoker	389 (28.6)	221 (32.5)	168 (24.7)	0.001
Chronic renal failure	11 (0.8)	3 (0.4)	8 (1.2)	0.130
History of CVA	39 (2.9)	13 (1.9)	26 (3.8)	0.035
History of MI	7 (0.5)	3 (0.4)	4 (0.6)	1.000^**†**^
Hypertension	322 (23.6)	138 (20.3)	184 (27.0)	0.003
COPD	59 (4.3)	28 (4.1)	31 (4.6)	0.690
Diabetes mellitus	155 (11.4)	72 (10.6)	83 (12.2)	0.348
CHF	25 (1.8)	11 (1.6)	14 (2.1)	0.545
Active cancer	13 (1.0)	6 (0.9)	7 (1.0)	1.000
Mental disorder	143 (10.5)	88 (12.9)	55 (8.1)	0.004
Head AIS*	3 (3–4)	3 (3–4)	3 (3–4)	1.000
Head AIS 3	782 (57.4)	391 (57.4)	391 (57.4)	1.000
Head AIS 4	330 (24.2)	165 (24.2)	165 (24.2)	
Head AIS 5	250 (18.4)	125 (18.4)	125 (18.4)	
ISS	16 (10–21)	16 (10–21)	16 (10–21)	0.547
ISS >15	695 (51.0)	344 (50.5)	351 (51.5)	0.704

Numbers may not add to 100% duet to missing values

Values are numbers (percentages) unless indicated otherwise, univariable analysis using Chi-square test unless indicated otherwise

*MVC* Motor vehicle crash, *MCC* Motor cycle crash, *AVP* Auto versus pedestrian, *CVA* Cerebrovascular accident, *MI* Myocardial infarction, *COPD* Chronic obstructive pulmonary disease, *CHF* Congestive heart failure, *AIS* Abbreviated injury scale, *ISS* Injury severity score

^*^Numbers are medians (interquartile range), univariable analysis using Mann–Whitney test

^†^Fishers exact test

In the matched cohort patients, the incidence of hypotension (SBP <90 mmHg) was similar between the groups (1.0% in the stimulants group vs 1.5% in the control group, *p* = 0.475) as was the incidence of GCS <9 (21.6% vs 21.9% respectively, *p* = 0.879). However, patients that tested positive for stimulants had a higher SBP [(142 (127–161) vs 138 (124–155) mmHg], were more frequently tachycardic (8.9% vs 5.9%).

The in-hospital mortality was 7.8% in the stimulant group and 7.9% in patients with no use of drugs or alcohol (*p* = 0.920). There was a trend toward a higher craniectomy rate in the stimulant group compared to the no drug/alcohol group (5.4% vs 3.5%; *p* = 0.089). In patients who required a craniectomy no difference was seen in the craniectomy timing [3.6 (1.6–11.4) hours in the stimulant group vs 4.0 (2.1–11.8) hours in the no drug/alcohol group, *p* = 0.507]. The ICP monitor rate was not significantly different between the two groups either [38 patients (5.6%) had an ICP monitoring in the stimulant group and 37 patients (5.4%) in the no drug/alcohol group, *p* = 0.905]. If mechanical ventilation was required (*p* = 0.338), there was a trend toward an increased ventilation duration in the stimulant group [4 (2–9) vs 3 (2–7) days, *p* = 0.078]. This trend toward an increased ventilation duration in the stimulant group was also observed in the subgroup of patients without a craniectomy [3 (2–8) vs 3 (2–6) days, *p* = 0.085]. The HLOS was significantly longer in patients with isolated stimulant use [5 (3–11) days] compared to those without any pre-injury drug or alcohol use [5 (3–9) days; *p* = 0.012]. The HLOS was 15 (7–26) days in patients who required a craniectomy and 5 (3–9) days in patients who had conservative treatment without craniectomy (*p* < 0.001). In patients who did not have a craniectomy HLOS was 5 (3–10) days in the stimulant group vs 5 (3–9) days in the no drug/alcohol group, *p* = 0.021. No difference was seen in the overall complication rate between patients under the influence of stimulants and those without drug/alcohol use (*p* = 0.223) (Table 2).

**Table 2:** Outcomes after matching

	All patients *N* = 1362 (%)	Stimulant only use *N* = 681 (%)	No drug/alc. use *N* = 681 (%)	*p*-value
Mortality in hospital	107 (7.9)	53 (7.8)	54 (7.9)	0.920
Mortality within 72 hrs	41 (3.1)	19 (2.8)	22 (3.3)	0.597
Craniectomy rate	61 (4.5%)	37 (5.4%)	24 (3.5%)	0.089
Time to craniectomy (hrs)*	3.9 (1.9–11.4)	3.6 (1.6–11.4)	4.0 (2.1–11.8)	0.507
Mechanical ventilation	438 (33.2)	234 (34.4)	204 (31.9)	0.338
Ventilator Days^**‡**^	3 (2–8)	4 (2–9)	3 (2–7)	0.078
Hospital LOS	5 (3–10)	5 (3–11)	5 (3–9)	0.012
	8.9 (STD 11)	9.5 (STD 12)	8.2 (STD 10)	
Complications				
Overall	57 (4.2)	33 (4.8)	24 (3.5)	0.223
Acute kidney injury	15 (1.1)	9 (1.3)	6 (0.9)	0.436
ARDS	4 (0.3)	3 (0.4)	1 (0.1)	0.624^**†**^
DVT	14 (1.0)	9 (1.3)	5 (0.7)	0.283
PE	6 (0.4)	3 (0.4)	3 (0.4)	1.000^**†**^
Thromboembolic events (DVT/PE)	19 (1.4)	11 (1.6)	8 (1.2)	0.488
Severe sepsis	4 (0.3)	3 (0.4)	1 (0.1)	0.624^**†**^
Myocardial infarction	1 (0.1)	0 (0.0)	1 (0.1)	1.000^**†**^
VAP	16 (1.2)	10 (1.5)	6 (0.9)	0.314
Stroke/CVA	7 (0.5)	4 (0.6)	3 (0.4)	1.000^**†**^

Numbers may not add to 100% duet to missing values

Values are numbers (percentages) unless indicated otherwise, univariable analysis using Chi-square test unless indicated otherwise

Craniectomy includes craniotomy procedures, *hrs* Hours, *LOS* Length of stay, *STD* Standard deviation, *ARDS* Acute respiratory distress syndrome, *DVT* Deep vein thrombosis, *PE* Pulmonary embolism, *VAP* Ventilator associated pneumonia, *CVA* Cerebrovascular accident

^*^Numbers are medians (interquartile range), univariable analysis using Mann–Whitney test

^**†**^Fishers exact test; mean and STD for hospital LOS reported as well

^**‡**^Only of the patients who were ventilated

### Adjusted effect of stimulant use in patients with isolated traumatic brain injury

Mortality was not independently associated with pre-injury stimulant use in isolated TBI (OR 0.95, 95% CI 0.61–1.49). However, the multivariable regression analysis in the matched cohort confirmed the significantly prolonged HLOS (RC 1.13, 95% CI 1.03–1.24) and the trend toward a higher craniectomy rate (OR 1.63, 95% CI 0.95–2.82) in the isolated stimulant group compared to no drug/alcohol group (Table 3).

**Table 3:** Adjusted effect of stimulant use as independent predictors of outcomes in comparison to no drug or alcohol use

Clinical outcome	Drug	adjusted *p*	RC	OR	95% CI for OR	
					Lower	Upper
Mortality^† a^	Stimulants	0.835		0.95	0.61	1.49
Craniectomy° ^b^	Stimulants	0.078		1.63	0.95	2.82
Mechanical ventilation* ^c^	Stimulants	0.264		1.16	0.90	1.50
Complications, overall^† d^	Stimulants	0.163		1.48	0.85	2.57
Hospital length of stay^‡ e^	Stimulants	0.008	1.13		1.03	1.24

Logistic regression analysis: checked for age, gender, mechanism of injury, overall comorbidities, hypotension, tachycardia, and head abbreviated injury score (AIS)

^a^Adjusted for age, mechanism of injury, overall comorbidities, hypotension, tachycardia, head AIS

^b^Adjusted for gender, overall comorbidities, head AIS

^c^Adjusted for age, mechanism of injury, overall comorbidities, hypotension, tachycardia, and head AIS

^d^Adjusted for mechanism of injury, hypotension, tachycardia, and head AIS

^e^Adjusted for mechanism of injury, tachycardia, and head AIS

^†^*χ*^2^ *p* = 0.589 Cox and Snell *R*^2^ = 0.128, Nagelkerke *R*^2^ = 0.302, AUROC = 0.85 (95% CI = 0.81–0.89)

°*χ*^2^ *p* = 0.176, Cox and Snell *R*^2^ = 0.063, Nagelkerke *R*^2^ = 0.205 AUROC = 0.83 (95% CI = 0.78–0.87)

^*^χ^2^ *p* = 0.741, Cox and Snell *R*^2^ = 0.179, Nagelkerke *R*^2^ = 0.249, AUROC = 0.76 (95% CI = 0.73–0.78)

^†^*χ*^2^ *p* = 0.892, Cox and Snell *R*^2^ = 0.023, Nagelkerke *R*^2^ = 0.080 AUROC = 0.71 (95% CI = 0.64–0.78)

^‡^ *R*^2^ = 0.090 and adjusted *R*^2^ = 0.87

The subgroup analyses to account for remaining differences in comorbidities generated similar results to the total cohort in both magnitude and direction (Supplemental Table 4).

Furthermore, regression analysis revealed no significant differences between patients under the influence of amphetamine/methamphetamine and those under the influence of cocaine. The reduced sample size as a result of splitting up the stimulants limited the statistical power, and no significant effect on outcomes was demonstrated compared to patients without illicit drug or alcohol use (Supplemental Table 5).

No significant collinearity was detected between the predictor variables of the regression models. The VIF was smaller than 1.5 for all variables included in regression models. Both the logistic and linear regression models fit the data well. Results of the model performance analysis are outlined in Table 3 and Supplemental Tables 4–5.

## Discussion

The present study was designed to evaluate pre-injury stimulant use in isolated severe blunt TBI among a large population in the United States. Our initial hypothesis that pre-injury stimulant use in isolated severe head injury is associated with worse outcomes compared to patients with no drug or alcohol use, was confirmed. Although we did not find an independent effect on mortality, pre-hospital stimulant use was independently associated with prolonged HLOS and a trend toward a higher craniectomy rate. In addition, there was also a trend toward an increased mechanical ventilation duration in the stimulant group when ventilation was required.

In general, the effect of pre-injury illicit drug use on outcomes is controversial and the reported results are often contradictory [11, 13–16, 19, 20]. Important factors that can confuse the interpretation of results are inclusion of all illicit drugs in one group, as well as the presence of multiple injuries. To eliminate these confounding factors, the present study focused on isolated severe TBI and stimulant drug use.

Only one study was found with an increased mortality associated with methamphetamine use [12]. This study retrospectively evaluated 4932 consecutive trauma patients who underwent toxicology screening at a level 1 trauma center during a 3-year period. Overall, 609 patients (12.3%) were found to be positive for methamphetamine. After adjusting for other drugs, ISS, age and sex, mortality was independently associated with methamphetamine use (OR 2.3 (95% CI 1.5–3.7). This study did not adjust for BAL or mechanism of injury, although there were significant differences between patients who tested positive and those who tested negative for methamphetamine. Furthermore, this study included a general trauma population, which may also explain how the results differed from our own.

A study performed at our institution in 2016 assessed pre-injury controlled substance use on clinical outcomes after trauma [21]. Overall, 10,166 patients (≥13 years old) who had a drug screen were included. The study population was analyzed according to: amphetamine, barbiturate, benzodiazepine, cocaine, opiate, PCP or no drug use. In the multivariable regression analysis adjusting for age, sex, SBP, GCS, ISS and mechanism of injury, pre-injury amphetamine use (OR 0.50, 95% CI 0.29–0.86) was independently associated with lower mortality. The protective effect on mortality was not shown for cocaine (OR 0.82, 95% CI 0.95–1.34). This study also included a general trauma population and did not specifically examine patients with TBI. In addition, more than 40% of the study population was not screened for alcohol, though positive alcohol results in the non-tested patients could bias the results. Furthermore, no specific corrections were made for patients with a positive BAL. These potential confounders and the different study population likely explain the reduced mortality in the amphetamine group when compared with our results.

Another study [22] recently published used the national trauma database (NTDB) to evaluate the effect of amphetamine and cocaine on mortality. This study evaluated 317,688 patients who underwent urine drug screening in 2017. In the multivariate analysis they adjusted for the following parameters: age, sex, race, cardiac and non-cardiac comorbidities, ISS, mechanism of trauma, trauma regions of head, chest, abdomen, and extremity, surgery, and emergency surgery. A protective association between cocaine and mortality [OR 0.9 (*p* = 0.028)] was found, however, not for amphetamine use [OR 0.97 (*p* = 0.405)]. For this study, the authors did not consider poly-drug or alcohol use, which may act as relevant confounders. Our data confirmed that a quarter of all patients who tested positive for an illicit drug also tested positive for an additional drug. Furthermore, the demographics between the stimulant positive and negative patients were significantly different. It is therefore possible that the protective effects observed with cocaine use may be a result of confounding variables, rather than based on a true pharmacologic effect.

Yeung et al. [20] aimed to determine the independent effect of cocaine use on mortality in severe TBI patients (head AIS >2). It is important to emphasize that this is the only study that focused specifically on patients with TBI. The study by Yeung et al. compared, retrospectively, 138 cocaine positive patients to 603 cocaine negative patients with severe TBI over a 4-year period. The multivariable regression analysis revealed that cocaine use (OR 0.33 CI 95% 0.04–2.7) was not independently associated with mortality. These results are in line with our findings, although the study by Yeung et al. [20] did not exclude severe associated injuries. Furthermore, Yeung and colleagues did not adjust for the concomitant use of other drugs or alcohol. As an example, in the cocaine positive group, 50.7% had a positive BAL compared to 37.8% in the cocaine negative group (*p* = 0.008). This inhomogeneous distribution of a positive BAL between the two groups may have acted as a confounder.

The concern that pre-injury stimulant use in patients with TBI affects patient physiology was confirmed in our study. Patients who were under the influence of prehospital stimulant use were more frequently tachycardic, had a higher SBP. Although there was a trend toward a higher craniectomy rate in patients using pre-injury stimulants, time to craniectomy was even shorter in the stimulant group. Therefore, the concern that pre-injury stimulant use may obscure diagnosis in the emergency bay, which could subsequently delay treatment and alter patient outcomes was not confirmed in the present study. This may be different in patients with an increased likelihood of hemorrhagic shock such as patients with severe abdominal or thoracic injuries. In this setting, it is conceivable that ostensibly compensated blood pressure due to the pre-injury stimulant use may delay the potentially life-saving laparotomy or thoracotomy.

The trend toward a higher craniectomy rate in patients who were under the influence of pre-injury stimulants was not expected. It can be speculated that the higher blood pressure in the stimulant group might aggravate the intracranial hematoma and consequently increase the craniectomy rate compared to patients without any drug or alcohol use.

Only a few studies examined secondary outcomes in trauma patients who were under the influence of pre-injury stimulants. In line with our findings, most of these studies reported that pre-injury stimulant use was associated with a longer hospital LOS [13, 19, 23, 24].

In our study the prolonged HLOS in the stimulant group may partly be explained by the trend toward higher craniectomy rate in this group, because patients who required a craniectomy had a significant longer HLOS (15 vs 5 days, *p* < 0.001). However, in patients without a craniectomy, HLOS was also prolonged in the stimulant group compared to the no drug/alcohol group (*p* = 0.021). Furthermore, the trend toward an increased ventilation duration in the stimulant group was also observed in the subgroup of patients without craniectomy. Difficulties in pain-management and drug tolerance including emerging withdrawal symptoms may contribute to the extended HLOS and the increased ventilation duration in the stimulant group, regardless of the craniectomy rate.

This large database study allowed for an evaluation of pre-injury stimulant use and its impact on outcomes in patients with isolated severe head injury. The analysis of patients with isolated stimulant use, as well as the exclusion of patients with a drug screening only positive due to administered drugs for treatment reduced potential confounders and allowed for a clean assessment. The 1:1 matching to patients with no drug or alcohol use, the additional adjustment in the regression analysis including the sub analyses performed, are definite strengths of the study. However, some study limitations should be acknowledged. Drug and alcohol screening are routinely ordered only for patients with altered mental status, for all other patients the screening is ordered at the discretion of the evaluating trauma team. Overall, only about a third of all patients included in 2017 TQIP database were fully screened for drugs and alcohol. Therefore, some patients who used drugs or alcohol may have not been captured in this study and a selection bias may be present. In addition, the TQIP database does not provide continuous drug levels. A dose-dependent relationship could therefore not be assessed. Also, the present study grouped together all stimulants, to increase the power of the study. The two largest groups, cocaine and amphetamine/methamphetamine, were evaluated in an additional regression analysis, which revealed no significant differences between these drugs. The small number of patients who were exclusively under the influence of ecstasy did not allow for a meaningful analysis, and differences with other stimulants could have been missed. However, the physiological effects of the summarized drugs are very similar and no significant differences are to be expected. Finally, the common complication of delirium in the presence of TBI and stimulant use could not be assessed in this study because the TQIP database does not provide any specific information on delirium rates.

Future research is warranted in TBI patients to better understand the effect of pre-injury stimulant use. Especially the trend toward a higher craniectomy rate in patients under stimulant use was not expected, and future research is warranted to better understand the underlying mechanisms. The study of biochemical and physiological changes under the influence of stimulants in patients with TBI may contribute to this understanding.

## Supplementary Information

Below is the link to the electronic supplementary material.

Supplementary file1 (DOCX 16 KB)

Supplementary file2 (DOCX 15 KB)

Supplementary file3 (DOCX 16 KB)

Supplementary file4 (DOCX 14 KB)

Supplementary file5 (DOCX 15 KB)
