# Supplementary material for: Pre-injury stimulant use in isolated severe traumatic brain injury: effect on outcomes
Source: Eur J Trauma Emerg Surg. 2022 Sep 6;49(4):1683–91. doi: 10.1007/s00068-022-02095-7 (PMC9446589; doi:10.1007/s00068-022-02095-7)
Supplement: Supplementary file 1 — Supplementary file1 (DOCX 16 KB) [file 68_2022_2095_MOESM1_ESM.docx]

|  | **All patients**  N=2,378 (%) | **Stimulant only use**  N=1,055 (%) | **No drug/alc. use**  N=1,323 (%) | ***p-value*** |
| --- | --- | --- | --- | --- |
| **Demographics** |  |  |  |  |
| Age* | 53 (34-68) | 46 (33-57) | 62 (38-77) | **<0.001** |
| ≥65 years | 673 (29.4) | 105 (10.0) | 568 (45.8) | **<0.001** |
| Gender, male | 1,679 (70.6) | 820 (77.7) | 859 (64.9) | **<0.001** |
|  |  |  |  |  |
| **Mechanism of Injury** |  |  |  |  |
| Fall | 1,148 (48.3) | 320 (30.3) | 828 (62.6) | **<0.001** |
| MVC | 410 (17.2) | 210 (19.9) | 200 (15.1) |  |
| MCC | 107 (4.5) | 75 (7.1) | 32 (2.4) |  |
| AVP | 223 (9.4) | 138 (13.1) | 85 (6.4) |  |
| Assault | 318 (13.4) | 224 (21.2) | 94 (7.1) |  |
| other | 172 (7.2) | 88 (8.3) | 84 (6.3) |  |
|  |  |  |  |  |
| **Comorbidities** |  |  |  |  |
| *Overall* | 1,488 (62.6) | 627 (59.4) | 861 (65.1) | **0.005** |
| Current smoker | 626 (26.3) | 408 (38.7) | 218 (16.5) | **<0.001** |
| Chronic renal failure | 26 (1.1) | 5 (0.5) | 21 (1.6) | **0.009** |
| History of CVA | 86 (3.6) | 15 (1.4) | 71 (5.4) | **<0.001** |
| History of MI | 12 (0.5) | 4 (0.4) | 8 (0.6) | **0.441** |
| Hypertension | 795 (33.4) | 204 (19.3) | 591 (44.7) | **<0.001** |
| COPD | 114 (4.8) | 41 (3.9) | 73 (5.5) | **0.064** |
| Diabetes mellitus | 337 (14.2) | 103 (9.8) | 234 (17.7) | **<0.001** |
| CHF | 103 (4.3) | 20 (1.9) | 83 (6.3) | **<0.001** |
| Active cancer | 56 (2.4) | 7 (0.7) | 49 (3.7) | **<0.001** |
| Mental disorder | 262 (11.0) | 156 (14.8) | 106 (8.0) | **<0.001** |
|  |  |  |  |  |
