# Supplementary material for: Pre-injury stimulant use in isolated severe traumatic brain injury: effect on outcomes
Source: Eur J Trauma Emerg Surg. 2022 Sep 6;49(4):1683–91. doi: 10.1007/s00068-022-02095-7 (PMC9446589; doi:10.1007/s00068-022-02095-7)
Supplement: Supplementary file 2 — Supplementary file2 (DOCX 15 KB) [file 68_2022_2095_MOESM2_ESM.docx]

|  | | | | | |
| --- | --- | --- | --- | --- | --- |
|  | **All patients**  N=2,378 (%) | **Stimulant only use**  N=1,055 (%) | **No drug/alc. use**  N=1,323 (%) | ***p-value*** |  |
| **Admission data** |  |  |  |  |  |
| Systolic blood pressure* | 142 (127-161) | 141 (127-159) | 143 (127-162) | **0.204** |  |
| Hypotension, | 28 (1.2) | 9 (0.9) | 19 (1.5) | **0.193** |  |
| Heart rate* | 86 (74-100) | 88 (76-103) | 84 (72-98) | **<0.001** |  |
| Tachycardia | 160 (6.8) | 88 (8.4) | 72 (5.5) | **0.005** |  |
| GCS* | 14 (10-15) | 14 (10-15) | 15 (11-15) | **0.021** |  |
| GCS <9 | 492 (21.2) | 214 (20.7) | 278 (21.7) | **0.584** |  |
|  |  |  |  |  |  |
| **Head AIS*** | 3 (3-4) | 3 (3-4) | 4 (3-5) | **<0.001** |  |
| Head AIS 3 | 1,245 (52.4) | 598 (56.7) | 647 (48.9) | **<0.001** |  |
| Head AIS 4 | 602 (25.3) | 283 (26.8) | 319 (24.1) |  |  |
| Head AIS 5 | 531 (22.3) | 174 (16.5) | 357 (27.0) |  |  |
|  |  |  |  |  |  |
| **Injury severities** |  |  |  |  |  |
| ISS* | 17 (10-24) | 16 (10-21) | 17 (10-25) | **0.079** |  |
| ISS >15 | 1,299 (54.6) | 545 (51.7) | 754 (57.0) | **0.009** |  |
|  |  |  |  |  |  |
