# Supplementary material for: Pre-injury stimulant use in isolated severe traumatic brain injury: effect on outcomes
Source: Eur J Trauma Emerg Surg. 2022 Sep 6;49(4):1683–91. doi: 10.1007/s00068-022-02095-7 (PMC9446589; doi:10.1007/s00068-022-02095-7)
Supplement: Supplementary file 3 — Supplementary file3 (DOCX 16 KB) [file 68_2022_2095_MOESM3_ESM.docx]

|  | **All patients**  N=2,378 (%) | **Stimulant only use**  N=1,055 (%) | **No drug/alc. use**  N=1,323 (%) | ***p-value*** |
| --- | --- | --- | --- | --- |
| **Mortality in hospital** | 241 (10.1) | 69 (6.5) | 172 (13.0) | **<0.001** |
| Mortality within 72hrs | 98 (4.2) | 25 (2.4) | 73 (5.7) | **<0.001** |
|  |  |  |  |  |
| **Craniectomy rate** | 91 (3.8%) | 54 (5.1%) | 37 (2.8%) | **0.003** |
| Time to craniectomy (hrs)* | 4.1 (1.9-12.8) | 4.0 (1.7-15.7) | 4.5 (2.1-10.9) | **0.198** |
|  |  |  |  |  |
| **Mechanical ventilation** | 774 (33.6) | 364 (34.6) | 410 (32.8) | **0.355** |
| Ventilator Day**^‡^** | 3 (2-8) | 4 (2-9) | 3 (2-8) | **0.005** |
|  |  |  |  |  |
| **Hospital LOS** | 5 (3-10) | 5 (3-11) | 5 (3-10) | **0.084** |
|  |  |  |  |  |
| **Complications** |  |  |  |  |
| *Overall* | 93 (3.9) | 50 (4.7) | 43 (3.3) | **0.063** |
| Acute kidney injury | 20 (0.8) | 10 (0.9) | 10 (0.8) | **0.610** |
| ARDS | 6 (0.3) | 5 (0.5) | 1 (0.1) | **0.094^†^** |
| DVT | 21 (0.9) | 13 (1.2) | 8 (0.6) | **0.104** |
| PE | 7 (0.3) | 3 (0.3) | 4 (0.3) | **1.000^†^** |
| Thromboembolic events (DVT/PE) | 26 (1.1) | 15 (1.4) | 11 (0.8) | **0.169** |
| Severe Sepsis | 7 (0.3) | 4 (0.4) | 3 (0.2) | **0.707^†^** |
| Myocardial infarction | 4 (0.2) | 0 (0.0) | 4 (0.3) | **0.134^†^** |
| *VAP* | 33 (1.4) | 20 (1.9) | 13 (1.0) | **0.059** |
| Stroke/CVA | 12 (0.5) | 5 (0.5) | 7 (0.5) | **0.850** |
|  |  |  |  |  |
