# Supplementary material for: Pre-injury stimulant use in isolated severe traumatic brain injury: effect on outcomes
Source: Eur J Trauma Emerg Surg. 2022 Sep 6;49(4):1683–91. doi: 10.1007/s00068-022-02095-7 (PMC9446589; doi:10.1007/s00068-022-02095-7)
Supplement: Supplementary file 4 — Supplementary file4 (DOCX 14 KB) [file 68_2022_2095_MOESM4_ESM.docx]

| **Adjusted for** | **Drug** | **adjusted p** | **OR** | **95% CI for OR** | |
| --- | --- | --- | --- | --- | --- |
|  |  |  |  | **Lower** | **Upper** |
| Current Smoker^†^ | Stimulants | 0.892 | 0.97 | 0.62 | 1.51 |
|  |  |  |  |  |  |
| Chronic renal failure° | Stimulants | 0.850 | 0.96 | 0.62 | 1.49 |
|  |  |  |  |  |  |
| History of CVA* | Stimulants | 0.991 | 1.00 | 0.64 | 1.56 |
|  |  |  |  |  |  |
| Hypertension^†^ | Stimulants | 0.743 | 0.93 | 0.60 | 1.45 |
|  |  |  |  |  |  |
| Mental disorder‡ | Stimulants | 0.905 | 0.97 | 0.63 | 1.52 |
|  |  |  |  |  |  |
