# Supplementary material for: Pre-injury stimulant use in isolated severe traumatic brain injury: effect on outcomes
Source: Eur J Trauma Emerg Surg. 2022 Sep 6;49(4):1683–91. doi: 10.1007/s00068-022-02095-7 (PMC9446589; doi:10.1007/s00068-022-02095-7)
Supplement: Supplementary file 5 — Supplementary file5 (DOCX 15 KB) [file 68_2022_2095_MOESM5_ESM.docx]

| **Clinical outcome** | **Drug** | **adjusted p** | **RC** | **OR** | **95% CI for OR** | |
| --- | --- | --- | --- | --- | --- | --- |
|  |  |  |  |  | **Lower** | **Upper** |
| Mortality^† a^ | Amphetamine/methamphetamine | 0.300 |  | 0.738 | 0.42 | 1.31 |
|  | Cocaine | 0.397 |  | 1.288 | 0.717 | 2.31 |
|  |  |  |  |  |  |  |
| Craniectomy° ^b^ | Amphetamine/methamphetamine | 0.222 |  | 1.52 | 0.78 | 2.95 |
|  | Cocaine | 0.772 |  | 1.12 | 0.51 | 2.45 |
|  |  |  |  |  |  |  |
| Mechanical ventilation* | Amphetamine/methamphetamine Cocaine | 0.666 0.442 |  | 1.07 1.14 | 0.78 0.81 | 1.48 1.61 |
|  |  |  |  |  |  |  |
| Complications, overall^† d^ | Amphetamine/methamphetamine | 0.135 |  | 1.64 | 0.86 | 3.12 |
|  | Cocaine | 0.348 |  | 1.42 | 0.69 | 2.92 |
|  |  |  |  |  |  |  |
| Hospital length of stay^‡ e^ | Amphetamine/methamphetamine | 0.110 | 1.09 |  | 0.98 | 1.21 |
|  | Cocaine | 0.791 | 1.02 |  | 0.91 | 1.14 |
|  |  |  |  |  |  |  |
